# 30 Year Patterns of Mortality in Tobago, West Indies, 1976-2005: Impact of Glucose Intolerance and Alcohol Intake

**DOI:** 10.1371/journal.pone.0014588

**Published:** 2011-01-25

**Authors:** Mariam Molokhia, Dorothea Nitsch, Alan Leslie Patrick, Paul McKeigue

**Affiliations:** 1 Division of Epidemiology and Population Health, London School of Hygiene and Tropical Medicine, London, United Kingdom; 2 Kavanagh Street Medical Centre, Port of Spain, Trinidad, West Indies; 3 Public Health Sciences/Molecular Medicine Centre, University of Edinburgh, Edinburgh, United Kingdom; Mayo Clinic College of Medicine, United States of America

## Abstract

**Objectives:**

To determine the main predictors of all-cause and cardiovascular (CV) mortality in a rural West Indian population in Plymouth, Tobago over 30 years.

**Methods:**

Questionnaire survey for CV risk factors and alcohol consumption patterns administered at baseline in 1976 with 92.5% response rate. 831/832 patients were followed up until 2005 or death.

**Results:**

Hypertension (>140/90 mm Hg) was prevalent in 48% of men and 44% of women, and 21% of men and 17% of women had diabetes. Evidence showed most predictors for all cause and cardiovascular mortality having the main effects at ages <60 years, (p-value for interaction<0.01) but no risk factors having sex-specific effects on mortality. The main predictors of all-cause mortality at age <60 years in the fully adjusted model were high sessional alcohol intake (hazard ratio (HR) 2.04, 95% CI 1.10-3.80), severe hypertension >160/95 mm Hg (HR 1.68, 95% CI 1.09-2.60), diabetes (HR 3.28, 95% CI 1.89-5.69), and BMI (HR 1.04, 95% CI 1.00-1.07). The main predictors of cardiovascular mortality were similar in the fully adjusted model: high sessional alcohol intake (HR 2.47 95% CI 1.10-5.57), severe hypertension (HR 2.78 95% CI 1.56-4.95), diabetes (HR 3.68 95% CI 1.77-7.67) and additionally LVH, (HR 5.54 95% CI 1.38-22.26), however BMI did not show independent effects. For men, high sessional alcohol intake explains 27% of all cause mortality, and 40% of cardiovascular mortality at age <60 yrs. In adults aged <60 years, the attributable risk fraction for IGT/Diabetes and all cause mortality and cardiovascular mortality is 28% in women vs. 11% in men, and 22% in women vs. 6% in men respectively.

**Conclusions:**

In this Afro-Caribbean population we found that a major proportion of deaths are attributable to high sessional alcohol intake (in males), diabetes, and hypertension and these risk factors primarily operate in those below 60 years.

## Introduction

Tobago is the smaller of the twin island state of Trinidad and Tobago and lies in the South Caribbean just north of Venezuela. A cross-sectional evaluation of the population of Black Rock, Plymouth was conducted in 1976–1978 to evaluate the predictors of all-cause and cardiovascular risk factors and alcohol consumption, and participants followed up until 2005. [1] At that time hypertension, diabetes and alcohol consumption in males were noted to be common in the population. It was shown then, for men that the highest correlation for systolic and diastolic blood pressure was with BMI; for women highest correlations for systolic and diastolic blood pressure were with post-load blood sugar and BMI respectively. [2] More recent studies in Trinidad with a similar population indicate high levels of cardiovascular risk factors persist such as obesity (30%) and WHR amongst office workers. [3] The aim of this analysis was to identify the main predictors of all-cause and cardiovascular mortality (including diabetes and IGT) by age-group and sex in a rural West Indian population in Plymouth, Tobago.

## Methods

### Study population

The total Afro-Caribbean adult population of certified age 20 years and over of a rural village on the north Western coast of the island (Black Rock, Plymouth) was evaluated from 1976–1978. The study comprised 832 participants, 349 (42%) men, and 483 (58%) women, and examined cardiovascular risk factors and alcohol consumption patterns. This village was selected because it was representative of the general population at the time the survey was conducted, had easy access, low migration rates and inhabitants showed willingness to participate in the study.

### Exposures

The questionnaire administered at baseline included history of hypertension, diabetes, chest pain, smoking, alcohol consumption, and symptoms of alcohol dependence (CAGE questionnaire). Alcohol intake was measured in units per week and sessional intake determined against a validated questionnaire. A sessional intake referred to consumption of alcohol in one “session” or sitting. High sessional intake was defined as ≥6 units per session. Blood pressure was measured with mercury sphygomanometer; using 2 nurses trained against an audiotape. Three blood pressure readings were taken and the average calculated. Hypertension was defined as systolic pressure 160 mmHg or diastolic 95 mmHg (severe) and systolic pressure of 140 mmHg or diastolic pressure of 90 mmHg (moderate). Glucose tolerance and IGT were determined from venous plasma glucose, with 75 g glucose load after an overnight fast using 1980 WHO criteria for diabetes and IGT. Cardiothoracic ratio (CTR) on chest X-ray was measured on a subset of 40% (333/832) of participants (approximately every 3^rd^ participant) 206/483 (43%) of females and 127/349 (36%) of males. The CTR measured was calculated from a vertical line from aorta – widest diameter of heart from the right atrium to centre and left ventricle to centre. Minnesota coding for the ECGs was done by 4 observers trained against the Minnesota standards, and the following ECG categories were defined: major Q waves (codes 1-1 or 1-2), tall left-sided R waves (codes 3-1 or 3-3); S-T depression (codes 4-1 to 4-3), T wave inversion (codes 5-1 to 5-4). Left ventricular hypertrophy/strain pattern was defined as tall R waves plus either S-T depression or T wave inversion. [Appendix S1]

### Follow up

Follow-up was undertaken through the local death registry and through visits to participants' homes. Cause of death was ascertained from death certificates supplemented with verbal autopsy interviews of the doctor and the family. All deaths from heart disease or cerebro-vascular disease were classified as cardiovascular. Vital status in 2005 was ascertained for 831 of the 832 cohort members and the precise date of death was ascertained for 307 of the 308 individuals who were established to have died.

This study was conducted according to the principles expressed in the Declaration of Helsinki. The study was approved by the Ministry of Health for Trinidad & Tobago. All patients provided written informed consent for the collection of samples and subsequent analysis.

### Statistical analyses

Baseline associations were carried out with chi-square tests for association and t-tests where appropriate. Survival analyses were carried out using time since enrolment to event adjusting for age and sex. Predictors of mortality were assessed by univariable and multivariable associations, from all causes and from cardiovascular disease and examined in Cox regression models. BMI as a categorical variable using the WHO cut-offs for normal weight, underweight and obesity showed poorer fit in the models, and therefore was entered as a continuous variable. Serum urate was also entered as a continuous predictor into the models. This analysis estimates the hazard ratio (ratio of hazards in exposed compared with unexposed individuals) associated with unit change in each risk factor. Analyses included testing for interactions such as age <60 and 60+ years and sex. Tests of the proportional-hazards assumption was carried out with Schoenfeld residuals derived from the final models, with no evidence of violation of proportionality. The attributable risk fraction for main risk factors were calculated as p(r-1)/[1+p(r–1)], where p is the prevalence of the risk factor and r is the fully adjusted hazard rate ratio associated with the risk factor where applicable in a specific gender and age-group. All analyses were carried out using Stata version 10.

## Results

### Baseline findings

There were 349 (42%) men, and 483 (58%) women. The response rate was 92.5% (832/899). The age distribution of the study population was similar in both sexes; mean [SD] age men 44.1 [17.0] years and mean age for women 43.4 [17.6] years. (Table 1) Alcohol intake was common both in men and women, but the total amount consumed by men significantly exceeded the amount consumed by women (11.3 units per week in men and 2.5 units per week in women; p<0.0001). Approximately 24% of men consumed the alcohol in a binge-drinking pattern with a very high sessional intake which was uncommon in women. (In the UK, the Office of National Statistics defines heavy drinking as eight or more units of alcohol per day for men, and six or more for women, now widely classified as “binge drinking”). Alcohol dependence as evaluated by the CAGE questionnaire was much more prevalent in men when compared to women (6% in men and 0.2% in women with CAGE score >2, p<0.0001). Smoking prevalence (ever) were 52.7% in men and 9.6% in women (p<0.001). The highest prevalence of male smokers was in the 55–64 year age group (66.7%), while for females it was in the 65+ year age group (9.3%). The average BMI was slightly higher in women (27.6 kg m^−2^) when compared to men (24.1 kg m^−2^) (P<0.0001).

**Table 1:** Baseline means and prevalence of risk factors by sex.

Risk factor	Men (349)	Women (483)
Age (years), mean ± sd	44.1±17.0	43.4±17.6
Alcohol use ever, % (n with risk factor/total)	85.7% (299/349)	70.6% (341/483)
Alcohol consumption (units/week), % (n with risk factor/total)	11.3±13.6	2.5±4.6
Alcohol high amount (≥6units/session)	23.5% (82/349)	2.1% (10/482).1
CAGE score >2, % (n with risk factor/total)	5.7% (20/348)	0.2% (1/482)
Ever smoked	52.7% (184/349)	9.6% (46/481)
BMI (kg m^−2^), mean ±sd	24.1±3.9	27.6±6.5
Systolic BP (mmHg), mean ±sd	132±22	132±26
Diastolic BP (mmHg), mean ±sd	86±13	85±14
Hypertension (>160/95 mmHg), % (n with risk factor/total)	24.9% (87/349)	26.1% (126/483)
Hypertension (>140/90 mmHg), % (n with risk factor/total)	47.9% (167/349)	44.1% (213/483)
Cardiothoracic ratio, mean ±sd	50.2±5.8	52.7±7.4
Tall R waves, % (n with risk factor/total)	33.9% (118/348)	9.5% (46/483)
S-T depression, % (n with risk factor/total)	3.4% (12/348)	5.0% (24/483)
T wave inversion, % (n with risk factor/total)	1.4% (5/349)	1.4% (7/483)
LVH/strain, % (n with risk factor/total)	2.6% (9/348)	2.3% (11/483)
Major Q waves, % (n with risk factor/total)	2.3% (8/348)	0.8% (4/479)
Plasma cholesterol (mmol/l), mean ±sd	5.02±1.25	5.22±1.38
Uric acid (micromol/l), mean ±sd	291±83	251±77
Fasting glucose (mmol/l), mean ±sd	5.04±1.99	5.21±2.39
Post-load glucose (mmol/l), mean ±sd	6.48±2.93	7.11±3.43
Impaired glucose tolerance, % (n with risk factor/total)	12.3% (43/349)	15.1% (73/483)
Diabetes, % (n with risk factor/total)	21.2% (74/349)	17.2% (83/483)

Impaired glucose tolerance was present in similar frequency in both men and women. Diabetes prevalence was high and comparable between the sexes, 74/349 (21%) in males and 83/483 in females (17%), respectively, p = 0.1. IGT was present in 12% men and 15% women. Mean [SD] blood pressures were similar for males and females: 132 [22] mm Hg in men and 132 [26] mm Hg in women for systolic blood pressure and 86 [13] mm Hg in men and 85 [14] mm Hg in women. Although absolute values for the prevalence of hypertension (>140/90 mmHg) appeared slightly higher in males 167/349 (48%) than observed for females 213/483 (44%), this was not significant p = 0.28). There was no evidence for a difference in prevalence of severe hypertension (>160/95 mmHg) between the sexes (25% in men and 26% in women respectively, p = 0.7), but there was evidence that tall R waves (associated with LVH) were more common in men (34%) than in women (10%) respectively, (p<0.0001). LVH together with strain (Minnesota code 3∶1or 3 and 4∶1-3 or 5∶1-4) was present in 2.6% men and 2.3% women, p = 0.6.

### Causes of death during follow-up

At the 30 year follow-up, of the respondents aged less than 60 years at presentation, 203/279 (73%) men and 294/380 (77%) women were still alive, and 76 men and 86 women had died. Of the respondents who were 60 years and over at presentation, 4/70 (6%) men and 15/103 (15%) women were alive, and 66 men and 88 women had died. Cardiovascular deaths accounted for 29/76 (38% male deaths) and 47/86 (55% female deaths) in the 20–59 year age group at presentation and 29/66 (44% male deaths) men and 52/88 (59% female deaths) in the 60 years and over age group. Cancer caused 49 deaths (18 women, 31 men). There were 102 who died from other causes (54 women, 48 men), and unknown causes of death were recorded for in total 8 study participants (3 women, 5 men) (Table 2).

**Table 2:** Vital status at 30-year follow-up by sex and age at baseline.

Age at baseline	20–59		60+	
	Men	Women	Men	Women
Alive	203	294	4	15
Dead	76	86	66	88
Cardiovascular	29	47	29	52
Cancer	15	12	16	6
Other cause	31	26	17	28
Unknown cause	1	1	4	2

“Other cause” category includes accidents, infections, injury diabetic complications HIV, liver cirrhosis and senility.

### All-cause mortality

Univariable analyses of main predictors of death were carried out and are presented in Table 3. We found evidence that all-cause mortality patterns with several risk factors varied by age-group, hence all the present analyses were stratified into those below 60 years of age, and those who are 60 years and above.

**Table 3:** Predictors of all-cause mortality: single-risk factor associations, stratified by age at baseline, and within strata adjusted for age in years and sex.

Age at baseline	20–59		60+	
	Hazard rate ratio	95% CI	Hazard rate ratio	95% CI
Sex (M/F)	1.02	0.72–1.45	2.28	1.61–3.22
BMI (kg m^−2^)	1.04	1.01–1.07	1.00	0.97–1.03
High alcohol intake ≥6 units per session adj for smoking	1.94	1.21–3.10	1.35	0.64–2.88
CAGE score >2 adj for smoking	2.87	1.41–5.84	3.25	0.99–10.74
Ever smoked	1.26	0.83–1.92	1.13	0.75–1.68
Systolic BP (mmHg)	1.02	1.02–1.03	1.00	1.00–1.01
Diastolic BP (mmHg)	1.03	1.01–1.04	1.01	1.00–1.02
Hypertension (>160/95)	2.14	1.46–3.13	1.38	0.97–1.94
Tall R waves	1.18	0.76–1.85	0.91	0.62–1.33
LV hypertrophy/strain	7.94	3.18–19.84	1.56	0.86–2.84
Cardiothoracic ratio	1.01	0.97–1.06	1.05	1.01–1.09
Serum total cholesterol (mmol/l)	1.10	0.97–1.26	0.98	0.84–1.15
Serum uric acid (micromol/l)	1.00	1.00–1.00	1.00	1.00–1.00
Glucose intolerance (IGT/diabetes vs normoglycaemic)	1.76	1.22–2.52	1.25	0.89–1.76

For example, there was strong evidence for effect modification between sex and age on all-cause mortality (p for interaction <0.01), with the hazard ratio (HR) for men versus women being 2.28 (95% confidence interval (CI) 1.61-3.22) at ages of 60 years and above, whilst there was no evidence for a difference between men and women for ages <60 years.

In those aged less than 60 years at baseline, predictors of all-cause mortality in single risk factor analyses (adjusted for age and sex) were BMI with HR 1.04 (95% CI 1.01–1.07) per kg m^–2^ increase, high sessional alcohol consumption (HR 1.94, 95% CI 1.21-3.10) and alcohol dependence as assessed by CAGE questionnaire score >2 (HR 2.87, 95% CI 1.41-5.84), both adjusted for smoking, systolic and diastolic blood pressure, severe hypertension ≥160/90 mmHg (HR 2.14, 95% CI 1.46–3.13), LV hypertrophy/strain (HR 7.94, 95% CI 3.18-19.84), IGT/diabetes (HR 1.76, 95% CI 1.22–2.52). Serum urate had no discernible effects on all-cause mortality in this younger sample. There was no evidence for an effect of smoking on all-cause mortality. For most of these variables, the univariable associations appeared weaker for those aged over 60 years at baseline.

Subsequent analyses looked at mutually adjusted (multivariable) effects of the significant risk markers on survival (Table 4). There was a prominent effect of increasing years of age from baseline in both groups with 7 and 11 percent increase in mortality risk per year of age in those aged below and above 60 years respectively. Male sex was a major predictor of all cause mortality at ages of 60 years and above, with approximately 4 times the mortality risk when compared to a woman of the same age (HR 4.36, 95% CI 2.03-9.35). In those aged less than 60 years at baseline, BMI was a risk factor for all-cause mortality (HR 1.04, 95% CI 1.00-1.07) whilst it was protective at older age when adjusted for the other prevalent risk factors (HR 0.93, 95% CI 0.86-1.00). Diabetes (HR 3.28, 95% CI 1.89-5.69) and high sessional intake of alcohol (HR 2.04, 95% CI 1.10-3.80) were major risk factors for death at younger age, whilst there was no evidence for these in adjusted models beyond age of 60 years.

**Table 4:** Multivariable analysis of all-cause mortality by age group.

	Hazard rate ratio	p-value	95% CI	
Aged <60 years				
Age (years)	1.07	<0.001	1.05	1.09
Sex (M/F)	1.12	0.43	0.68	1.88
BMI (kg m^−2^)	1.04	0.03	1.00	1.07
Hypertensive ≥160/95 mmHg	1.68	0.02	1.09	2.60
LVH/strain	3.34	0.07	0.90	12.41
IGT	1.50	0.10	0.92	2.44
Diabetes	3.28	<0.001	1.89	5.69
High alcohol sessional intake ≥6 units per session	2.04	0.02	1.10	3.80
Ever smoked	0.93	0.80	0.55	1.59
CAGE >2	1.11	0.87	0.34	3.65
Aged >60 years*				
Age (years)	1.11	<0.001	1.06	1.17
Sex (M/F)	4.36	<0.001	2.03	9.35
Cardiothoracic ratio	1.08	0.01	1.02	1.14
BMI	0.93	0.04	0.86	1.00
CAGE >2	9.29	0.12	0.55	157.43

Each model includes age, sex, smoking, cholesterol, serum urate, LVH, IGT/Diabetes, alcohol intake, known cardiovascular risk factors and any other variables significant at p<0.05.

*>60 also adjusted for cardiothoracic ratio.

In those aged >60 years at baseline, the main predictor of all-cause mortality in multivariable analyses was the cardiothoracic ratio (HR 1.08 (95% CI 1.02-1.14)).

In the multivariable analyses there was statistical evidence for interaction of age-group with systolic blood pressure (p for interaction <0.0001), LVH/strain (p for interaction  = 0.02), and high sessional alcohol intake (p = 0.02), because effects of these variables appeared weaker in those with older age on the multiplicative scale that is underlying a Cox-model. In other words effects of risk factor and age are largely additive. For other variables, which on univariable analyses appeared to have higher point estimates in the older age-group when compared to the younger age-group below 60 years of age there was no evidence to support that the effects were indeed stronger, except for male sex.

### Cardiovascular mortality

As seen for all-cause mortality, we found that there was evidence for effects differing by age-group, hence we present the analyses stratified into those <60 and 60+ years of age. (Table 5)

**Table 5:** Predictors of cardiovascular mortality: single-risk factor associations, stratified by age at baseline, and within strata adjusted for age in years and sex.

Age at baseline	20–59		60+	
	Hazard rate ratio	95% CI	Hazard rate ratio	95% CI
Sex (M/F)	0.74	0.46–1.18	1.61	1.01–2.59
BMI (kg m^−2^)	1.03	1.00–1.08	1.03	1.00–1.07
High alcohol intake ≥6 units per session adj for smoking	2.27	1.23–4.18	1.01	0.30–3.39
CAGE score >2 adj for smoking	3.16	1.21–8.24	5.03	1.13–22.32
Ever smoked	1.29	0.74–2.25	0.83	0.47–1.49
Systolic BP (mmHg)	1.03	1.02–1.04	1.01	1.00–1.01
Diastolic BP (mmHg)	1.04	1.02–1.06	1.01	1.00–1.03
Hypertension (≥160/95 mmHg)	3.06	1.88–4.97	1.62	1.02–2.58
Tall R waves	1.52	0.86–2.69	0.77	0.45–1.32
LV hypertrophy/strain	17.14	6.59–44.59	1.06	0.42–2.62
Cardiothoracic ratio	1.01	0.96–1.07	1.06	1.01–1.11
Serum total cholesterol (mmol/l)	1.17	0.99–1.37	0.99	0.82–1.20
Glucose intolerance(IGT/diabetes vs. normoglycaemic)	1.62	1.01–2.59	1.49	0.94–2.34

In those aged less than 60 years at baseline, predictors of cardiovascular mortality in univariable analyses (adjusted for age and sex) were BMI with HR 1.03 (95% CI 1.00-1.08) per kg m^−2^ increase, high alcohol consumption (adjusted for smoking) (HR 2.27, 95% CI 1.23-4.18) and CAGE questionnaire score >2 with HR adjusted for smoking of 3.16 (95% CI 1.21-8.24), hypertension ≥160/90 mmHg HR 3.06 (95% CI 1.88–4.97), LVH/strain HR 17.14 (95% CI 6.59-44.59), IGT/diabetes HR 1.62 (95% CI 1.01-2.59). There was evidence in the fully adjusted models that high sessional alcohol intake had a weaker multiplicative effect in those aged 60+ years when compared to younger ages (p-value for interaction <0.0001) as well as systolic BP (p-value for interaction <0.0001) and LVH/strain (p-value for interaction = 0.01).

In those aged 60 years and more, male sex (HR 1.61, 95% CI 1.01-2.59), BMI (HR 1.03 (95% CI 1.00–1.07)), CAGE score >2, adjusted for smoking (HR 5.03, 95% CI 1.13-22.32), hypertension ≥160/95 mmHg (HR 1.62 (95% CI 1.02–2.58)), enlarged CTR (HR 1.06, 95% CI 1.01–1.11) were also associated with increased cardiovascular mortality.

In multivariable adjusted analyses, the associations with cardiovascular mortality were generally similar to the adjusted associations observed for all-cause mortality (Table 6). Older age was a major risk factor, and above the age of 60 years male sex. Being markedly hypertensive ≥160/95 mmHg at an age <60 years almost tripled the cardiovascular risk (HR 2.78, 95% CI 1.56-4.95), and having developed a left-ventricular strain led to an even higher risk of dying of cardiovascular disease (HR 5.54, 95% CI 1.38-22.26), even when adjusted for the other risk factors. Having diabetes at an age <60 years was independently associated with a HR of 3.68 (95 CI 1.77-7.67) of dying from cardiovascular disease. High sessional alcohol intake, adjusted for smoking led to a roughly 2.5 increased risk of dying from cardiovascular disease (HR 2.47, 95% CI 1.10-5.57), independently of blood pressure or other risk factors in those below 60 years. In the fully adjusted model, there was no apparent age & sex interaction (p-value for interaction = 0.12). In those aged over 60 years at baseline, age and sex remained the largest determinants of cardiovascular mortality.

**Table 6:** Multivariable analysis of cardiovascular mortality by age-group.

	Hazard rate ratio	p-value	95% CI	
Aged <60				
Age	1.06	<0.001	1.03	1.09
Sex (M/F)	0.75	0.42	0.37	151
BMI (kg m^−2^)	1.02	0.32	0.98	1.07
Hypertensive ≥160/95 mmHg	2.78	0.001	1.56	4.95
LVH/strain	5.54	0.02	1.38	22.26
IGT	1.35	0.36	0.71	2.56
Diabetes	3.68	<0.001	1.77	7.67
High sessional alcohol intake ≥6 units per session	2.47	0.03	1.10	5.57
Ever smoked	0.97	0.94	0.48	2.00
CAGE >2	1.02	0.98	0.24	4.28
Aged >60*				
Age	1.11	<0.001	1.04	1.18
Sex (M/F)	3.51	0.01	1.35	9.12
BMI (kg m^−2^)	0.97	0.53	0.90	1.06
Hypertension ≥160/95 mmHg	1.96	0.16	0.77	4.96
Cardiothoracic ratio	1.06	0.11	0.99	1.13
Ever smoked	0.89	0.81	0.34	2.31
High sessional alcohol amount ≥6 units per session	0.50	0.53	0.34	2.31

Each model includes age, sex, smoking, cholesterol, serum urate, LVH, IGT/Diabetes, alcohol intake, known cardiovascular risk factors and any other variables significant at p<0.05.

*>60 also adjusted for cardiothoracic ratio.

### Attributable risk fraction

The fractions of mortality attributable to hypertension and glucose intolerance were examined in those aged under 60 years at baseline by calculating the attributable risk fractions (ARF) for these two dichotomous risk factors adjusted for all cardiovascular risk factors including smoking for both sexes separately. Because high sessional alcohol intake is a risk factor that is largely prevalent in men, the sex-specific attributable risk fractions are very different, although the association of a given risk factor with mortality was found to be the same for both men and women and only differs by age (as in the analyses presented above). For men, high sessional alcohol intake explains 26.5% of deaths of any cause, and 39.9% of cardiovascular deaths at age <60 years, whilst in women the attributable risk fraction for alcohol is negligible. For hypertension, the attributable fractions of mortality from all causes and from cardiovascular disease at the level of 160/95 was more common amongst men (17.9%, 36.8%) when compared to women (7.5%, 11.5%) respectively. When fitting sex-specific models, the fractions of all-cause mortality attributable to glucose intolerance or diabetes in those aged <60 years at baseline were higher for all cause mortality in women, 28.3% compared to 10.9% in men. This sex difference was a consequence of the higher hazard rate ratios associated with glucose intolerance in women compared with men because deaths in men were driven by their alcohol intake a risk factor which was largely absent in women. Similarly, for cardiovascular mortality the ARF attributable to IGT/DM was 22.1% for women but and 5.8% for men [Appendix S2].

## Discussion

We have evaluated the effect of prevalent risk factors on mortality in this population over a 30 year follow-up. We found that excessive sessional alcohol consumption was 10 times higher in men when compared to women and that this explained 40% of cardiovascular deaths of men <60 years. Because high sessional alcohol consumption is very rare in women, the major risk factor in women is impaired glucose tolerance and diabetes, which explained 28% of deaths in women of ages <60 years.

Studies in Tobago have identified alcohol as a possible risk factor for cardiovascular disease, attributable to direct effects of alcohol on the heart, but also through effects on blood pressure. (1) A study in Korea found a strong effect of binge-drinking on haemorrhagic stroke in both men and women. [4] Similar patterns as in this study have been found in Russia, where research has also shown that it is unlikely that recorded cardiovascular deaths are misreported. [5]

### Sex differentials in mortality

A remarkable finding in this study was that in those aged less than 60 years at baseline, there was no sex difference in all-cause mortality rates or cardiovascular mortality rates. Excess mortality in men compared with women was observed only in participants aged 60 years and over at baseline. This contrasts with the experience of developed countries where excess mortality in younger men compared with women is consistently observed in all ethnic groups including African-Americans. [6] Part of the explanation may be that in societies where there is a large male excess in adult mortality, this is largely attributable to injuries and to coronary heart disease, both of which were rare in this rural Caribbean population. [7] Another part of the explanation appears to be the high prevalence of diabetes in this population where roughly a fifth of the population suffers from manifest diabetes, which is the highest prevalence of any country in the Americas. [7] The mortality rates are more than 3 times increased for diabetics when compared to others and diabetes may attenuate the female advantage in cardiovascular mortality [8]. The blood glucose levels of this population are similar to those of those of the other Caribbean islands, [9] and similar associations with all-cause, cardiovascular and cerebrovascular mortality have been found in the St James Survey. [10]

The St James Survey [10] has shown that hypertension is a major problem in both main ethnic groups in Trinidad and prevalence in all Caribbean populations appears to have increased. [11] In our population most hypertensive individuals were not on any medication at the time of the interview. [2] Elevated systolic blood pressure has been shown to be an independent and strong predictor of risk of cardiovascular and renal disease [12]–[13], We found hypertension and hypertension related ECG manifestations when present at younger age were significant mortality predictors. Control of hypertension should be a priority in this population, particularly in those with diabetes [14]–[16] or ECG evidence of LV hypertrophy/strain. [17]

Although in those aged below 60 years at baseline, rates of severe hypertension are similar in males (16.9%) and females (16.6%), the ARF for all cause and cardiovascular mortality is over twice as high in males.

Mean CT ratios are higher than that of a similar population in Wales, (1) and increased with hypertension. (2) Elevated CT ratio was associated with a greatly increased all cause and cardiovascular mortality particularly in those below 60 years at baseline. ECG findings compatible with ischaemia were uncommon in both sexes. [9] This is compatible with studies in the UK showing relatively low rates of coronary disease among Caribbean migrants, and with the St James Survey showing low rates of coronary disease among people of West African descent. [10] However ECG findings suggestive of LV hypertrophy were relatively common in this population, 2.6% in males and 2.2% in females. This has been substantiated by echocardiography in this population [9] with equal prevalence in men and women. [18] Our findings support the aggressive screening and control of cardiovascular risk factors at ages <60 years to prevent heart failure and death in this high-risk population.

Diabetes was a significant predictor of all cause mortality in the <60 age group (fully adjusted model) HR 3.28 (95% CI 1.89-5.69) but not in the older age group. Results were similar for cardiovascular mortality in the <60 age group HR 3.68 (95% CI 1.77-7.67). Studies in Afro-Caribbeans have also demonstrated increased cardiovascular risk in those with IGT compared to Europeans. [19] Again, this finding supports screening at a younger age to prevent diabetes related deaths in this population.

Although body mass index was a weak predictor of mortality in those aged less than 60 years this population, both the two main risk factors (hypertension and glucose intolerance) were strongly related to body mass index. BMI was strongly predictive of hypertension, as in other Caribbean populations, [20] although recent reports suggest that WHR may be a more accurate predictor of cardiovascular mortality in other populations. [21] The importance of obesity as a risk factor for mortality (we showed protective effect of higher BMI in adjusted analyses of all-cause mortality in ages >60 years) is likely to be underestimated in this study because of weight loss among individuals who are in poor health and BMI at older age is likely to capture muscle mass rather than fat. [22]

A potential limitation of this study is that this was a cohort consisting of a single village in Tobago. However, there was almost complete follow-up on all participants of the original survey. As the cross-sectional study relied on ECG findings and X-ray dimensions and did not store bloods or collect urinary markers, we were unable to perform analyses of more novel cardiovascular risk factors or look at the effects of kidney function or proteinuria on long-term outcome in this population.

In summary, in this population, high sessional alcohol intake, glucose intolerance and diabetes, as well as hypertension are the strongest predictors of mortality, particularly in the 20–59 age group. The relationship of obesity to mortality is likely to be underestimated in this study, as independent data on WHR were not available. The high mortality among women is partly explained by glucose intolerance/diabetes and to a lesser degree, the effects of hypertension. The strong association of mortality with ECG evidence of LV hypertrophy/strain is consistent with the importance of hypertension as a risk factor, particularly in those with diabetes or ECG evidence of LV hypertrophy/strain. Data also suggest that individuals with high sessional alcohol intake may have independently increased risk for all cause and cardiovascular mortality, as these associations remain after adjusting for smoking.

There are clear public health benefits for modifiable risk factors (such as alcohol and obesity) for all cause and cardiovascular mortality in this population which may also apply to Afro-Caribbean migrants overseas. This suggests a need for evaluation of different public health intervention programs in this region to address modifiable risk factors to improve the adverse risk profile of this population. It would be useful to explore more sensitive measures of body fat distribution (WHR) and renal function (estimated glomerular filtration rate, GFR, micro and macro albuminuria) in relation to long-term all cause and cardiovascular mortality.

### Conclusions

We have identified in this Afro-Caribbean population that a major proportion of deaths are attributable to high sessional alcohol intake, diabetes, and hypertension and that these risk factors primarily operate in those with younger age. These findings may also have implications for early education and suggested lifestyle modifications for Afro-Caribbeans overseas. Control of obesity would help to reduce the risk of developing diabetes and deliver benefits for those with hypertension, but lifestyle interventions to control obesity, although potentially amenable to public health intervention, are difficult to sustain in the long term.

## Supporting Information

Appendix S1 Risk factors for all cause and cardiovascular mortality.
(0.05 MB DOC)

Appendix S2 Attributable Risk Fraction (ARF) for all cause and cardiovascular mortality.
(0.04 MB DOC)
