# Supplementary material for: 30 Year Patterns of Mortality in Tobago, West Indies, 1976-2005: Impact of Glucose Intolerance and Alcohol Intake
Source: PLoS One. 2011 Jan 25;6(1):e14588. doi: 10.1371/journal.pone.0014588 (PMC3026774; doi:10.1371/journal.pone.0014588)
Supplement: Appendix S1 — Risk factors for all cause and cardiovascular mortality. (0.05 MB DOC) [file pone.0014588.s001.doc]

| **Risk factor** | **How measured** | **Potentially modifiable** |
| --- | --- | --- |
| Alcohol use ever, % (n with risk factor /total) | Binary Y/N | Yes |
| Alcohol consumption (units/week) , % (n with risk factor /total) | Units | Yes |
| Alcohol high amount (≥6units/session) | Numbers of units in one drinking session | Yes |
| CAGE score > 2, % (n with risk factor /total) | Screening test for alcohol dependence based on 4 questions ; >2 indicative of alcohol dependence | Yes |
| Ever smoked | Y/N | Yes |
| BMI (kg m^-2^), mean ± sd | Body Mass Index continuous | Yes |
| Systolic BP (mmHg) , mean ± sd | Blood pressure continuous | Yes |
| Diastolic BP (mmHg) , mean ± sd | Blood pressure continuous | Yes |
| Hypertension (>160/95 mmHg), % (n with risk factor /total) | Binary severe hypertension cut off | Yes |
| Hypertension ( >140/90 mmHg), % (n with risk factor /total) | Binary moderate hypertension cutoff | Yes |
| Cardiothoracic ratio, mean ± sd | Maximum transverse diameter of the heart divided by the greatest internal diameter of the thoracic cage (inside to inside of rib)  Normal is usually less than 50% |  |
| Tall R waves, % (n with risk factor /total) | ECG tall R waves may reflect left ventricular hypertrophy (R wave greater than 25mm in V5, V6) | Yes |
| S-T depression, % (n with risk factor /total) | ECG ST segment depression can reflect cardiac ischaemia | Yes |
| T wave inversion, % (n with risk factor /total) | ECG T wave inversion may indicate acute MI | No |
| LVH/strain, % (n with risk factor /total) | Left Ventricular Hypertrophy is thickening of the left ventricular heart muscle and may be caused by cardiac disease or hypertension | Yes |
| Major Q waves, % (n with risk factor /total) | Pathologic major Q waves are a sign of previous myocardial infarction | No |
| Plasma cholesterol (mmol/l) , mean ± sd | Measures blood cholesterol | Yes |
| Uric acid (micromol/l) , mean ± sd | Putative risk factor for CHD and diabetes | No |
| Fasting glucose (mmol/l), mean ± sd | Measures fasting blood glucose | Yes |
| Post-load glucose (mmol/l), mean ± sd | Measures glycaemic clearance | Yes |
| Impaired glucose tolerance* (mmol/L), % (n with risk factor /total) | Impaired glucose tolerance; ≥7.8 mmol/L may be precursor to diabetes | Yes |
| Diabetes*, % (n with risk factor /total) | According to post load glucose ≥11.1mmol/L or previously diagnosed diabetic | No |

Minnesota Code Classification System used for Electrocardiographic Findings

*1980 WHO Criteria
