# Supplementary material for: 30 Year Patterns of Mortality in Tobago, West Indies, 1976-2005: Impact of Glucose Intolerance and Alcohol Intake
Source: PLoS One. 2011 Jan 25;6(1):e14588. doi: 10.1371/journal.pone.0014588 (PMC3026774; doi:10.1371/journal.pone.0014588)
Supplement: Appendix S2 — Attributable Risk Fraction (ARF) for all cause and cardiovascular mortality. (0.04 MB DOC) [file pone.0014588.s002.doc]

|  |  | **All-cause mortality** | | **Cardiovascular mortality** | |
| --- | --- | --- | --- | --- | --- |
|  | **Prevalence** | **Age-adjusted hazard rate ratio*** | **Attributable risk fraction** | **Age-adjusted hazard rate ratio*** | **Attributable risk fraction** |
| ***Hypertension* (160/95)** |  |  |  |  |  |
| Men | 16.9% | 2.3 | 17.9% | 4.3 | 36.8% |
| Women | 16.6% | 1.5 | 7.5% | 2.7 | 11.5% |
| **Glucose intolerance/Diabetes** |  |  |  |  |  |
| Men | 32.2% | 1.4 | 10.9% | 1.6 | 5.8% |
| Women | 28.6% | 2.4 | 28.3% | 2.0 | 22.1% |
| ***High alcohol intake (per session)*** adjusted for smoking |  |  |  |  |  |
| Men | 26.5% | 2.4 | 26.6% | 3.5 | 39.9% |
| Women | 2.6% | 0.6 | - | 2.1 | - |

*adjusted for age, hypertension, IGT/diabetes, alcohol intake, LVH, BMI, smoking, urate, cholesterol (and CAGE score)
